# Pediatric Patients and Tonometers

**DOI:** 10.4274/tjo.44778

**Published:** 2017-08-15

**Authors:** Sora Yasri, Viroj Wiwanitkit

**Affiliations:** 1 KMT Primary Care Center, Bangkok, Thailand; 2 DY Patil University Faculty of Medicine, Pune, India

**Keywords:** Pediatric, Tonometer, measurement

## Dear Editor,

The recent publication on “Pediatric Patients and Tonometers” is interesting.^1^

Eraslan et al.^1^ concluded that “Because Tono-Pen (TP) measurements were lower than Goldmann applanation tonometer (GAT) measurements and non-contact tonometer measurements were higher than GAT measurements, patient follow-ups, treatment strategies, and surgery plans must be organized taking these differences into consideration”. The results in this report are similar to a recent report by Galgauskas et al.^2^ In fact, the use of different kinds of tonometer can result in different measures values and this has to be kept in mind by practitioners. The correlation study can be useful for checking the variability of the tool. Nevertheless, there are some concerns that should be addressed. First, the lack of a gold standard for the comparative study is a big issue for further discussion. At present, we can only perform the inter-tool agreement check but there is no gold standard for checking the accuracy of the measurement. Second, for each tool the within-day and between-day precision of the tool should also be checked. Finally, the calibration error of the tool should be regularly checked since it can contribute to incorrect measurement results.^3^ In the present report by Eraslan et al.,^1^ there is no error checking as well.
