# Supplementary material for: Pediatric Patients and Tonometers
Source: Turk J Ophthalmol. 2017 Aug 15;47(4):247–8. doi: 10.4274/tjo.44778 (PMC5563557; doi:10.4274/tjo.44778)
Supplement: Related texts

# Reply to the Authors

**Authors:** Muhsin Eraslan, Eren Çerman, Sena Sümmen

## Dear Editor,

We are grateful to Yasri and Wiwanitkit for their interest in our work and their valuable comments. The authors kindly reminded that the use of different kinds of tonometer can result in different measured values, which corresponds to the conclusion of our study. They mentioned the lack of a gold standard in intra ocular pressure measurements but as all we know, the Goldmann applanation tonometer (GAT) is defined as the gold standard measurement method in a large number of studies.^1^ As they emphasized in their letter, the findings of our study are similar to a recent report of Galgauskas et al.^2^ where GAT is defined as the gold standard. But as Garcia Feijoo et al.^3^ mentioned in their study, despite GAT being the gold standard for determining intra ocular pressure since the last century, the substantial effects of several ocular variables such as axial length, curvature, rigidity, and corneal thickness are its obvious limitations. Yasri and Wiwanitkit also mentioned that we can only perform the inter-tool agreement and they recommended that a correlation study can be useful for checking the variability of the tool. However, to evaluate whether the differences between two measurements of the same variable are significant, previous studies recommend studying the differences, not the agreement.^4^ The correlation shows the relationship between one variable and another, not the differences, and it is not the best technique for assessing the comparability between methods.^4^ Bland-Altman (B-A) plots compare two clinical measurements that each provide some errors in their measure and these plots are extensively used to evaluate the agreement between two different instruments or two measurement techniques.^5^ B-A plot analysis can also be used for assessing the comparability between a new measurement technique or method with a gold standard, as even a gold standard does not-and should not-imply it to be without error.^6^ These analyses evaluate a bias between the mean differences and estimate an agreement interval, within which fall 95% of the differences of the second method compared to the first one. It is common to compute 95% limits of agreement for each comparison (average difference ±1.96 standard deviation (SD) of the difference). The compared methods can be used interchangeably unless the differences within mean ±1.96 SD are clinically important.^4^

In our study, Pearson’s test was used to determine the presence of correlations. Differences of 1.96 SD from the mean were used when calculating the limits of agreement. Associations between differences and means were analyzed using B-A plots. This was mentioned in the third paragraph of the Materials and Methods section of our study.

Within-day and between-day precision was checked for each tool and because our GAT and Tonopen are older than 1 year, the calibration error of the tools is routinely checked on a daily basis and the non-contact tonometer was calibrated once a month as recommended by the manufacturers. These were also mentioned in the second paragraph of the Materials and Methods section of our study. This is consistent with the study of Choudhari et al.^7^, which was mentioned by Yasri and Wiwanitkit in their letter; they concluded that GATs older than a year should be checked at least monthly. Therefore, we believe that all of the abovementioned limitations are important but it is unlikely that they affected our results significantly.

## References

[1] Steinberg J, Mehlan J, Frings A, Druchkiv V, Richard G, Katz T, Linke SJ (2015). Pachymetry and Intraocular Pressure Measurement by Corneal Visualization Scheimpflug Technology (Corvis ST): A Clinical Comparison to the Gold Standard. Ophthalmologe..

[2] Galgauskas S, Strupaite R, Strelkauskaite E, Asoklis R (2016). Comparison of intraocular pressure measurements with different contact tonometers in young healthy persons. Int J Ophthalmol..

[3] Garcia-Feijoo J, Martinez-de-la-Casa JM, Morales-Fernandez L, Saenz Frances F, Santos-Bueso E, Garcia-Saenz S, Mendez-Hernandez C (2015). New Technologies for Measuring Intraocular Pressure. Prog Brain Res..

[4] Giavarina D (2015). Understanding Bland Altman Analysis. Biochem Med (Zagreb)..

[5] Hanneman SK (2008). Design, Analysis, and Interpretation of Method-Comparison Studies. AACN Adv Crit Care..

[6] Bland JM, Altman DG (1999). Measuring Agreement in Method Comparison Studies. Stat Methods Med Res..

[7] Choudhari NS, Rao HL, Ramavath S, Rekha G, Rao A, Senthil S, Garudadri CS (2016). How Often the Goldmann Applanation Tonometer Should be Checked for Calibration Error?. J Glaucoma..
